# Asian-White disparities in short sleep duration by industry of employment and occupation in the US: a cross-sectional study

**DOI:** 10.1186/1471-2458-14-552

**Published:** 2014-06-03

**Authors:** Chandra L Jackson, Ichiro Kawachi, Susan Redline, Hee-Soon Juon, Frank B Hu

**Affiliations:** 1 Department of Nutrition, Building II, Room 302, Harvard School of Public Health, 655 Huntington Avenue, Boston, MA, USA; 2 Department of Social and Behavioral Sciences, Harvard School of Public Health, Boston, MA, USA; 3 Department of Medicine, Brigham and Women’s Hospital and Beth Israel Deaconess Medical Center, Harvard Medical School, Boston, MA, USA; 4 Department of Health, Behavior & Society, The Johns Hopkins Bloomberg School of Public Health, Baltimore, MD, USA

**Keywords:** Sleep, Work, Industry, Occupation, Asian, Race

## Abstract

**Background:**

Although short sleep is associated with an increased risk of morbidity as well as mortality and has been shown to vary by industry of employment and occupation, little is known about the relationship between work and sleep among Asian Americans.

**Methods:**

Using a nationally representative sample of US adults (n = 125,610) in the National Health Interview Survey from 2004–2011, we estimated prevalence ratios for self-reported short sleep duration (<7 hours) in Asians compared to Whites by industry of employment and occupation using adjusted Poisson regression models with robust variance.

**Results:**

Asians were more likely to report short sleep duration than Whites (33 vs. 28%, p < 0.001), and the Asian-White disparity was widest in finance/information and healthcare industries. Compared to Whites after adjustments, short sleep was also more prevalent among Asians employed in Public administration (PR = 1.35 [95% CI: 1.17,1.56]), Education (PR = 1.29 [95% CI: 1.08,1.53]), and Professional/Management (PR = 1.18 [95% CI: 1.03,1.36]). Short sleep, however, was lower among Asians in Accommodation/Food (PR = 0.81 [95% CI: 0.66, 0.99]) with no difference in Retail. In professional and support-service occupations, short sleep was higher among Asians, but was not different among laborers.

**Conclusions:**

U.S. Asian-White disparities in short sleep varied by industries, suggesting a need to consider both race and occupational characteristics to identify high-risk individuals.

## Background

Insufficient sleep (<7 hours/day) has been shown to increase risk of weight gain and obesity, hypertension, diabetes, coronary heart disease and subsequent mortality [1-11]. Among Asian populations in the US and abroad, short sleep is independently associated with insulin resistance [12] and an increased risk of diabetes [13]. In 2008, Asian Americans had a higher age-adjusted prevalence of diabetes (8.2%) than Whites (7.0%) [14], and for any given weight, they also appear to have a higher risk of obstructive sleep apnea compared to Whites [15]. In a meta-analysis of prospective studies of sleep duration and mortality, both short and long sleep in East Asian countries (Japan, Taiwan) were more strongly associated with mortality compared to studies conducted in Europe and the US [16]. In a nationally representative sample of the US, Asians, however, reported the least sleep complaints compared to Latinos, Blacks and Whites in a study that found lower socioeconomic status (SES) was associated with higher sleep complaints [17]. While Asian Americans tend to have high educational attainment and to be well represented in professional occupations with relatively high incomes, there may also be important variation in short sleep by occupation within the Asian population and in comparison to Whites.

Short sleep duration has been shown to vary by industry and occupation among US workers with certain industries (e.g. transportation, manufacturing, public administration) well above the median and several (e.g. education, agriculture) well below. [18,19]. There, however, have been limited race-specific investigations of sleep by industry of employment and occupation although important racial/ethnic differences in influential factors are likely to exist. For instance, one’s race/ethnicity as well as occupation likely plays an important role in producing psychosocial stress and job strain that negatively impacts health through, for example, discrimination or limited control over job demands/prestige as illustrated by the Karasek and Theorell demand-control model [20-22]. In a previous study, we found that the prevalence of short sleep increased as professional responsibility increased among Blacks while the prevalence decreased among their White counterparts [23]. We concluded that Black-White disparities in sleep duration by industry and occupation may reflect racial differences in work schedules as well as stressors and stress associated with specific jobs. In particular, Blacks are more likely to engage in shift work (especially night shifts) with non-standard work schedules and to work multiple low-wage jobs [24,25]. Blacks are also more likely to have long work hours, report job stress related to discrimination, and to work in low control/high demand positions with low decision-making power. Among professional workers, Blacks, may have more limited networks to provide supportive resources, compared to Whites, and may develop an extraordinarily high work ethic that could damage health through inadequate sleep as a coping strategy to overcome negative racial stereotypes/stressors [26-28].

The impact of industries of employment and occupations on sleep among Asian Americans as well as how they may be affected differently than Whites and Blacks is important to identify and understand to create effective, tailored interventions to improve sleep for optimal health and productivity in this population. However, very few studies have investigated Asian-White disparities in the work-sleep relationship that may occur due to differences in, for example, SES, work ethic and drive to succeed, social support, cultural factors like religion, and acculturation. Therefore, we sought to examine racial/ethnic disparities in short sleep duration by industry of employment and occupation using a nationally representative sample of US Asian and White adults reporting short sleep in the National Health Interview Survey from 2004 to 2011.

## Methods

### The National Health Interview Survey

We analyzed data from the National Health Interview Survey (NHIS), which is a series of cross-sectional, nationally representative surveys that use a three-stage stratified cluster probability sampling design to conduct in-person interviews in the households of non-institutionalized US civilians. A detailed description of NHIS procedures has been previously published [29]. In short, an annual probability sample of households was interviewed by trained interviewers from the US Census Bureau on a continuous basis throughout the year to obtain information about health and other characteristics of each member of the sampled household. The data were collected using computer-assisted personal interviewing (CAPI). A randomly selected adult and child (not used in this analysis) provided more extensive health-related information, and the final response rate for sample adults was 67% (range: 61-72%). Our study was approved by the Harvard School of Public Health’s Institutional Review Board, and the NHIS received informed consent from each study participant.

### Study participants

Non-Hispanic White and Non-Hispanic Asian (hereafter, White and Asian) adults aged ≥18 years were included in our study. Participants were excluded from the study analysis if they had missing data on sleep, industry and employment status, were deemed unemployed or not in the labor force, or had an extreme body-mass index (BMI) – i.e. either <15 or >70 kg/m^2^. Although previous studies suggest sleep patterns among immigrants may differ from individuals born in the US [30], we included non-US born participants for evaluation and robust sample size (particularly, among the Asian participants). As NHIS is not designed to provide accurate estimates of military persons, participants in armed forces were excluded. Our final sample consisted of 125,610 adults.

### Variable measurements

#### Sleep duration

Participants reported the average hours of sleep they usually get in a 24-hour period. Interviewers were trained to report hours of sleep in whole numbers, rounding values of 30 minutes or more up to the nearest hour or otherwise rounding down. Short sleep duration was defined as usual sleep duration of <7 hours, and adequate sleep was categorized as 7 hours of sleep. Seven hours of sleep was used as the reference because it has been shown to be associated with the lowest levels of morbidity and mortality [7,11,31], and our sample size could provide stable estimates. We are comparing short and adequate sleepers only, and do not note differences among longer sleepers as the causes (e.g. depression, poor health status, low socioeconomic status) have been shown to fundamentally differ from short sleep and the potential mechanisms linking long sleep to poor health outcomes are considered more speculative.

#### Race/ethnicity

Race/ethnicity was based on self-identification. Participants were asked, ‘What race or races do you consider yourself to be?”, They then selected 1 or more of the following categories: White, Black/African American, Asian, American Indian/Alaskan native or multiple race. The Asian category consists of ‘Filipino’ (24%), ‘Chinese’ (20%), ‘Asian Indian’ (20%), and ‘Other Asian’ (36%); sample size precluded us from further stratifying them by specific ethnic groups. We focus on Asian-White disparities in sleep duration because the underlying biological and social mechanisms are likely to further vary for other races/ethnicities. We have previously reported on Black-White disparities, and Whites are used as the comparison group for statistical stability and because this group represents the majority population in this country.

#### Industry of employment

For employed sample adults, the North American Industrial Classification System (NAICS) Codes were categorized into the following 8 industry categories: 1) ‘Construction’; ‘Manufacturing’; ‘Agriculture, Forestry, Fishing, and Hunting’; ‘Mining’; ‘Utilities’; and ‘Wholesale Trade’; and ‘Transportation and Warehousing’, 2) ‘Retail Trade’, 3) ‘Information’; ‘Finance and Insurance’; and ‘Real Estate and Rental and Leasing’, 4) ‘Professional, Scientific, and Technical Services’; ‘Management of Companies and Enterprises’; and ‘Administrative and Support and Waste Management and Remediation’, 5) ‘Education Services’, 6) ‘Health Care and Social Assistance’, 7) ‘Accommodation and Food Services’ as well as 8) ‘Other Services (except Public Administration)’; ‘Public Administration’; and ‘Arts, Entertainment, and Recreation’.

#### Occupation

Adults who were either working at a paying or non-paying job during the week prior to the survey, who had a job or business but were not at work during the prior week, or who ever worked were asked about their occupation, which was categorized based on the Standard Occupational Classification System. Based on type of work, we combined occupation categories into ‘Professional/management’, ‘Support Services’ and ‘Laborers’.

#### Covariates

Educational attainment was categorized as less than high school (<HS) (no high school diploma), high school (HS) (high school or general equivalency diploma), and greater than high school (>HS) (education beyond high school). Household income was dichotomized at above and below $35,000, and poverty status was based on being below the poverty line after the participants’ best estimate of total income of all family members from all sources before taxes. Employment status was based on the week prior to the interview for all adults, and was categorized as ‘working for pay’, ‘working without pay’, ‘job not at work’, ‘unemployed’, and ‘not in the labor force.’ Class of work (based on current, longest held, or most recently held job or work situation) was classified as either 1) an employee of a private company, business, or individual for wages, salary, or commission; 2) a federal, state, or local government employee; 3) self-employed in OWN business, professional practice or farm; 4) or working without pay in a family-owned business or farm.

Height and weight, based on self-report, were used to calculate body mass index (BMI) by dividing measured weight in kilograms by height in meters squared. In Whites, obesity was defined as BMI ≥30 kg/m^2^, overweight as 25.0 – 29.9 kg/m^2^, normal weight as 18.5 – 24.9 kg/m^2^, and underweight as BMI < 18.5 kg/m^2^. In Asians, obesity was defined as BMI ≥27.5 kg/m^2^, overweight as 23.0 – 27.4 kg/m^2^, normal weight as 18.5 – 22.9 kg/m^2^, and underweight as BMI < 18.5 kg/m^2^[32]. Marital status was classified as married/living with partner, divorced/separated/widowed, or never married, and both smoking status and lifetime alcohol consumption was categorized as ‘never’, ‘current’, or ‘former’. Leisure-time physical activity was categorized as ‘never/unable’, ‘low’, or ‘high’. Participants reporting ‘never’ or ‘unable to do this type activity’ were categorized as ‘none,’ and those engaging in at least some level of activity and providing a specific number of activity bouts were dichotomized at the midpoint of these bouts and labeled as ‘low’ or ‘high’. In terms of medical conditions, adults reported if they had ever been told by a doctor or other health professional that they had “hypertension, also called high blood pressure” or, separately, if they had “diabetes or sugar diabetes”. Participants were also asked if a doctor or other health professional ever diagnosed them as having any kind of heart condition or disease other than coronary heart disease, angina pectoris, or a myocardial infarction as well as if a doctor or other health professional ever diagnosed them as having coronary heart disease. These variables were combined to adjust for heart disease. Residential regions of the country were categorized as the South, Midwest, Northeast, and West, and participant self-reported general health status was considered excellent/very good, good, or fair/poor.

### Statistical analysis

We pooled NHIS data across 8 survey years (2004–2011), which was merged by the Integrated Health Interview Series [33]. Sampling weights that account for the unequal probabilities of selection resulting from the sample design, non-response, and oversampling of certain subgroups were employed in all analyses, and Taylor series linearization was used to calculate standard errors for variance estimation [34]. The STATA “subpop” command was used for correct variance estimation of estimates, and different sampling designs in 1997 to 2005 versus 2006 to 2008 were accounted for by the Integrated Health Interview Series. Rao-Scott Second-order corrected Pearson statistics take survey weights into account for contingency table chi-square tests [35]. Continuous variables were presented as means ± standard errors (SE), and categorical variables as absolute values with percentages. We used STATA statistical software version 12 (STATA Corporation, College Station, Texas, USA, 2007) [36].

We used Poisson regression models with a robust variance estimator to directly estimate prevalence ratios with corresponding 95% confidence intervals for short sleep duration in Asians compared to short sleep in Whites by industry of employment and, separately, for occupation [37]. Demographic, health behavior, socioeconomic, and clinical characteristics were pre-specified and entered into the model as groups in a stepwise manner. For greater statistical stability for the Asian-White comparisons, Whites were used as the reference categories because they had the largest sample size. For models stratified for Asians and Whites, we adjusted first for age in 3 categories (18–49, 50–64, 65+ years), and then for demographic factors such as sex, marital status, and educational attainment. Subsequently, we adjusted for health behaviors including smoking status, alcohol consumption, and leisure-time physical activity and then, in a separate model, we adjusted for self-reported health status, hypertension, diabetes, heart disease, cancer and 4 standard BMI categories. Living in poverty, household income above or below $35,000, classes of occupation as well as occupation (when investigating industry differences) were all accounted for in the final model. We used Rao-Scott second-order corrected Pearson statistics again for each industry to test for race-specific temporal trends in short sleep duration over the study period by industry of employment. In addition to testing racial disparities in short sleep duration for each survey period, differences in linear trends in short sleep from 2004 to 2011 between Asians and Whites within each industry category were formally tested using multivariable-adjusted linear regression models where survey year was treated as a dummy variable. In a subsidiary analysis, we investigated differences in short sleep prevalence by immigrant status.

## Results

### Study population

Our sample consisted of 125,610 (8,390 Asian; 117,220 White) participants. Their mean age was 51 ± 11 years, 51% were men, 5% were Asian, 32% (31 for Whites; 53 for Asians) had at least a college education. Among all participants, 35,961 (28%) were considered short sleepers (<7 hours), 40,409 (33%) adequate sleepers (7 hours), and 49,240 (39%) reported sleeping more than 7 hours. Table 1 shows weighted estimates of age-adjusted prevalence of short sleep by sociodemographic, health behavior and clinical factors among Asian and White participants. Asians were more likely to report short sleep than Whites (31 vs. 28%, p <0.001). For education, the greatest prevalence of short sleep was among high school graduates (36%) in Whites and in individuals with some college for Asians (36%). Short sleep prevalence in individuals living in poverty was similar for both Asians and Whites (35 vs. 37%). The overall percentage point difference in short sleep between Asians and Whites was 3%, 6% for professional/management positions, 6% for support services and 2% for laborers. Additional file 1: Table S1 shows the distribution of the aforementioned characteristics among participants with short sleep. Although the sample size was too low to stratify all analyses by Asian subgroup, the overall prevalence of short sleep duration varied by Asian subgroup with Chinese (prevalence (p) = 23.6% [95% CI: 21.0-26.4]) and Asian Indians (p = 24.8% [95% CI: 21.9-27.9]) having a significantly lower prevalence than Filipinos (p = 37.4% [95% CI: 34.7-40.1]) and Other Asians (p = 33.1% [95% CI: 31.0-35.2]).

**Table 1:** Age-adjusted prevalence of short sleep duration by sociodemographic, health behavior and clinical characteristics among 125,610 US Asian and White participants, 2004-2011

	**Short sleep duration (<7 hours)**					
	**White (n)**	**White (%)**	Asian*(n)	**Asian (%)**	**Total (n)**	**Total (%)**
		**95% CI**		**95% CI**		**95% CI**
**Sample size, short sleepers**	33,354	28 (27.8-28.5)	2,607	33 (31.0-34.1)	35,961	28 (27.9-28.6)
**Age group, (%)**						
18-49	18,172	31 (30.6-31.6)	1,611	29 (27.5-31.0)	19,783	31 (30.5-31.5)
50-64	9,346	29 (28.7-29.9)	637	36 (33.2-39.4)	9,983	30 (29.0-30.2)
≥65	5,836	21 (20.6-21.7)	359	32 (28.7-35.3)	6,195	22 (20.9-22.1)
**Women**	16,041	28 (27.9-28.8)	1,315	32 (30.2-34.4)	18,605	28 (28.0-28.9)
**Men**	17,313	28 (27.3-28.3)	1,292	33 (30.7-34.8)	17,356	28 (27.5-28.4)
**Educational attainment**						
<High school	10,418	31 (30.0-31.3)	459	32 (28.5-35.1)	10,877	31 (30.0-31.2)
High school graduate	3,303	36 (34.3-36.8)	202	32 (27.6-36.8)	3,505	35 (34.0-36.5)
Some college	11,145	30 (29.1-30.4)	651	36 (32.4-38.6)	11,796	30 (29.3-30.5)
≥ College	8,488	23 (22.1-23.2)	1,295	32 (29.5-33.9)	9,783	23 (22.7-23.8)
**Marital status**						
Married	15,624	26 (25.8-26.7)	1,416	32 (29.8-33.5)	17,040	27 (26.1-27.0)
Divorced/separated/widowed	10,868	35 (34.4-35.8)	479	37 (33.9-40.5)	11,347	35 (34.4-35.8)
Never married	6,791	28 (26.6-28.6)	708	34 (29.3-37.9)	7,499	28 (26.8-28.8)
**Non-US born**	1,400	27 (25.8-28.8)	1,873	31 (29.5-32.7)	3,273	29 (27.7-29.8)
**Living in poverty**	3,526	37 (35.5-38.3)	297	35 (29.9-40.3)	3,823	37 (35.4-38.1)
**Household income < $35,000**	18,223	27 (26.1-27.0)	1,570	32 (30.5-34.4)	19,793	27 (26.4-27.2)
**Class of worker**						
Private wage	24,910	29 (28.7-29.6)	1,989	33 (31.3-35.1)	26,899	29 (28.8-29.7)
Government	5,286	26 (25.3-26.9)	396	31 (26.7-35.5)	5,682	26 (25.5-27.1)
Self employed	2,970	25 (24.1-26.0)	200	29 (24.9-33.7)	3,170	25 (24.2-26.2)
**Occupation**						
Professional/management	6,345	25 (24.6-26.1)	705	31 (27.7-33.9)	7,050	26 (24.9-26.3)
Support services	14,848	27 (26.0-27.0)	1,196	33 (30.7-34.9)	16,044	27 (26.3-27.3)
Laborers	12,034	32 (31.7-33.0)	689	34 (31.1-36.4)	12,723	32 (31.8-33.0)
**Occupation [work hours (≥40 hours/wk)]**						
Professional/management	4,234	27 (25.6-28.0)	515	37 (31.1-42.0)	4,749	27 (26.0-28.4)
Support services	6,750	28 (27.0-28.9)	685	37 (33.1-41.7)	7,435	29 (27.5-29.4)
Laborers	5,643	33 (31.8-34.4)	345	35 (29.6-40.7)	5,988	33 (32.0-34.6)
**Industry**						
Manufacturing/construction	10,628	30 (29.9-31.1)	594	31 (28.6-34.2)	11,222	30 (29.9-31.0)
Retail	3,763	29 (28.3-30.3)	244	32 (27.7-36.0)	4,007	29 (28.4-30.3)
Finances/information	2,940	26 (24.7-26.5)	272	38 (32.0-43.9)	3,212	26 (25.2-27.0)
Profess/admin/man	3,116	26 (25.2-27.5)	331	30 (25.5-35.3)	3,447	26 (25.4-27.6)
Education	2,814	23 (21.9-23.7)	246	29 (24.7-34.2)	3,060	23 (22.1-23.9)
Health care	4,201	29 (27.8-29.8)	426	37 (33.0-40.7)	4,627	29 (28.3-30.1)
Accommodation and food	2,043	33 (31.5-35.3)	185	28 (22.7-32.8)	2,228	33 (30.9-34.6)
Public administration, arts	3,849	27 (26.4-28.3)	309	34 (31.1-37.5)	4,158	28 (26.7-28.6)
**Health behaviors**						
Smoking status						
Never	15,572	26 (25.2-26.2)	1,855	32 (30.4-34.3)	17,427	26 (25.6-26.5)
Current	8,433	28 (27.2-28.5)	395	33 (30.0-36.6)	8,828	28 (27.4-28.6)
Former	9,317	34 (33.3-34.9)	354	34 (28.8-38.4)	9,671	34 (33.3-34.8)
Alcohol consumption						
Never	3,799	27 (25.8-27.8)	739	31 (28.2-33.6)	4,538	27 (26.2-28.0)
Current	19,289	27 (26.7-27.6)	1,142	34 (31.9-36.9)	20,431	27 (26.8-27.8)
Former	4,878	32 (31.2-33.0)	236	38 (33.0-43.2)	5,114	32 (31.3-33.2)
Leisure-time physical activity						
Never/unable	11,542	31 (30.8-32.1)	868	33 (31.1-35.6)	12,410	31 (30.8-32.1)
Low	10,778	27 (26.0-27.2)	925	33 (29.9-35.7)	11,703	27 (26.2-27.4)
High	10,917	27 (26.0-27.1)	809	32 (28.9-34.4)	11,726	27 (26.2-27.3)
**Clinical characteristics**						
Overweight/Obese^a^	21,921	30 (29.4-30.3)	1,688	36 (33.1-37.9)	23,087	30 (29.5-30.4) ^c^
Obese^b^	10,153	33 (32.0-33.3)	714	38 (32.4-43.2)	10,457	33 (32.2-33.5) ^c^
Hypertension (yes)	10,901	32 (31.1-32.4)	704	37 (34.0-40.6)	11,605	32 (31.3-32.6)
Diabetes (yes)	2,892	33 (31.8-34.7)	217	33 (28.0-38.3)	3,109	33 (31.8-34.6)
Heart disease (yes)	4,469	33 (31.6-33.7)	186	42 (35.8-48.9)	4,655	33 (31.8-33.9)
Cancer (yes)	3,467	31 (29.4-31.9)	94	30 (23.5-37.2)	3,561	31 (29.4-31.9)
**Health status**						
Excellent/very good	18,680	25 (24.4-25.2)	1,531	31 (28.9-33.1)	20,211	25 (24.6-25.4)
Good	9,203	31 (30.5-31.9)	781	34 (31.3-36.5)	9,984	31 (30.6-31.9)
Fair/poor	5,453	40 (38.8-40.9)	293	42 (37.3-47.3)	5,746	40 (38.9-40.9)
**Region of country**						
Northeast	6,204	30 (28.9-30.6)	472	33 (29.4-36.4)	6,676	30 (29.1-30.7)
Midwest	9,823	28 (27.7-29.3)	352	34 (29.0-38.6)	10,175	29 (27.7-29.3)
South	11,234	28 (27.8-29.0)	488	32 (27.9-35.7)	11,722	28 (27.8-29.1)
West	6,093	25 (24.7-26.2)	1,295	32 (30.3-34.6)	7,388	26 (25.5-26.9)

Weighted estimates; n (%).

^a^Overweight/Obese defined by Body Mass Index ≥25 kg/m^**2**^ for Whites and ≥23 kg/m^**2**^ for Asians.

^b^Obesity defined by Body Mass Index ≥30 kg/m^**2**^ for Whites and ≥27.5 kg/m^**2**^ for Asians.

^c^Race-specific BMI standards were applied.

*Asian subgroups: Chinese (short sleep prevalence (p) = 23.6% [95% CI: 21.0-26.4]), Asian Indians (p = 23.6% [95% CI: 21.0-26.4]), Filipinos (p = 37.4% [95% CI: 34.7-40.1]) and Other Asians (p = 33.1% [95% CI: 31.0-35.2]).

### Asian-White differences in sleep duration by industry and occupation

Table 2 shows adjusted prevalence ratios of short sleep duration for Asians and Whites by industry of employment. Compared to Whites, adjusted short sleep was more prevalent in Asians employed in the following industries: Finances/Information/Real estate (prevalence ratio (PR) = 1.46 [95% confidence interval (CI): 1.26,1.69]), Health care and social services (PR = 1.39 [95% CI: 1.22,1.57]), Public administration/Other services (PR = 1.35 [95% CI: 1.17,1.56]), Education (PR = 1.29 [95% CI: 1.08,1.53]), Professional/Administrative/Management (PR = 1.18 [95% CI: 1.03,1.36]), and Manufacturing/Construction (PR = 1.14 [95% CI: 1.03,1.26]). Short sleep prevalence, however, was lower among Asians compared to Whites in the Accommodation and food services industry (PR = 0.81 [95% CI: 0.66, 0.99]). There was no observed difference between Asians and Whites in Retail (PR = 1.05 [95% CI: 0.87, 1.26]).

**Table 2:** Adjusted prevalence ratios of short sleep duration for Asians compared to Whites by industry of employment, National Health Interview Survey, 2004–2011 (n = 35,961)

	**Model 1:**	**Model 2:**	**Model 3:**	**Model 4:**	**Model 5:**
	**Age**	**Demographics**	**Health behaviors**	**Medical conditions**	**Occupational characteristics**
**Manufacturing/construction**	1.00	1.03	1.16	1.14	1.14
	(0.92-1.08)	(0.95- 1.12)	(1.06-1.28)	(1.04-1.26)	(1.03-1.26)
**Retail**	1.01	1.03	1.16	1.13	1.05
	(0.88-1.16)	(0.90-1.18)	(0.98-1.36)	(0.97-1.33)	(0.87-1.26)
**Finances/information**	1.30	1.36	1.49	1.44	1.46
	(1.14-1.48)	(1.19-1.54)	(1.29-1.72)	(1.25-1.65)	(1.26-1.69)
**Profess/admin/man**	0.97	1.01	1.16	1.14	1.18
	(0.86-1.09)	(0.90-1.14)	(1.02-1.33)	(1.00-1.30)	(1.03-1.36)
**Education**	1.20	1.20	1.27	1.25	1.29
	(1.04-1.37)	(1.04-1.38)	(1.08-1.49)	(1.06-1.47)	(1.08-1.53)
**Health care and social services**	1.21	1.26	1.42	1.40	1.39
	(1.10-1.34)	(1.13-1.39)	(1.27-1.60)	(1.25-1.57)	(1.22-1.57)
**Accommodation and food**	0.82	0.80	0.95	0.94	0.81
	(0.70-0.96)	(0.68-0.94)	(0.79-1.15)	(0.79-1.13)	(0.66-0.99)
**Public administration, arts**	1.19	1.20	1.38	1.37	1.35
	(1.06-1.34)	(1.07-1.35)	(1.22-1.57)	(1.21-1.56)	(1.17-1.56)

Model 1 adjusted for age categories.

Model 2 adjusted Model 1 + sex, marital status, educational attainment.

Model 3 adjusted Model 2 + smoking status, alcohol consumption, physical activity.

Model 4 adjusted Model 3 + health status, body mass index, hypertension, diabetes, heart disease, cancer.

Model 5 adjusted Model 4 + class of occupation, occupation, living in poverty, household income.

Adjusted prevalence ratios of short sleep duration for Asians compared to Whites by occupation are provided in Table 3. The prevalence of short sleep among Asians was higher among professional (PR: 1.25 (95% CI: 1.14-1.38) and management (PR: 1.28 (95% CI: 1.18-1.38) workers, and short sleep was not different among laborers (PR: 1.07 (95% CI: 0.97-1.18). Although limited by sample size, US-born Asians in professional occupations (PR: 1.56 (95% CI: 1.33-1.83) had a higher short sleep prevalence than Whites while non-US born Asians did not (see Additional file 2: Table S2).

**Table 3:** Adjusted prevalence ratios of short sleep duration for Asians compared to Whites by occupation, National Health Interview Survey, 2004–2011 (n = 35,961)

	**Model 1:**	**Model 2:**	**Model 3:**	**Model 4:**	**Model 5:**
	**Age**	**Demographics**	**Health behaviors**	**Medical conditions**	**Occupational characteristics**
**Professional/management**	1.08	1.11	1.25	1.23	1.25
	(0.99-1.17)	(1.02-1.20)	(1.14-1.37)	(1.13-1.35)	(1.14-1.38)
**Support services**	1.15	1.18	1.32	1.29	1.28
	(1.08-1.22)	(1.10-1.25)	(1.23-1.42)	(1.20-1.39)	(1.18-1.38)
**Laborers**	1.01	0.99	1.12	1.11	1.07
	(0.93-1.09)	(0.92-1.07)	(1.03-1.22)	(1.02-1.21)	(0.97-1.18)

Model 1 adjusted for age categories.

Model 2 adjusted Model 1 + sex, marital status, educational attainment.

Model 3 adjusted Model 2 + smoking status, alcohol consumption, physical activity.

Model 4 adjusted Model 3 + health status, body mass index, hypertension, diabetes, heart disease, cancer.

Model 5 adjusted Model 4 + class of occupation, living in poverty, household income.

### Trends in sleep duration by industry

**Figure 1:**
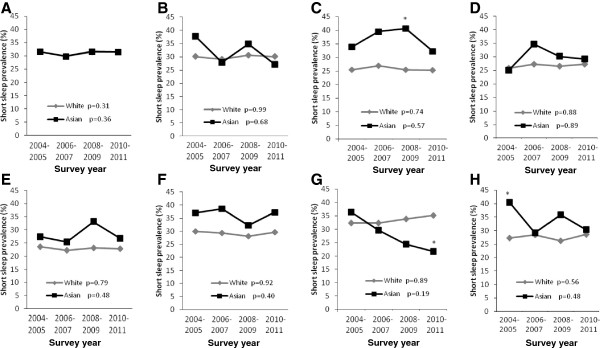
**Trends in the age-adjusted prevalence of short sleep duration by industry of employment among Asians and Whites, National Health Interview Survey, 2004-2011. A**. Manufacturing; Construction; Transportation; Wholesale trade; Agriculture; Utilities; Mining [*P* for interaction: 0.65]; lines are indistinguishable between Whites and Asians. **B**. Retail trade [*P* for interaction: 0.76]. **C**. Finance and insurance; Information; Real estate [*P* for interaction: 0.15]. **D**. Professional; Administrative; Management [*P* for interaction: 0.78]. **E**. Education [*P* for interaction: 0.53]. **F**. Health care and Social Assistance [*P* for interaction: 0.04]. **G**. Accommodation and Food services [*P* for interaction: 0.08]. **H**. Public Administration; Other services; Arts and Entertainment [*P* for interaction: 0.53] *p*-values in figure legends represent *p* for trends.

Figure 1 illustrates temporal trends in the age-adjusted prevalence of short sleep duration by industry of employment among Asians and Whites for each year from 2004 to 2011. Although all trends were statistically insignificant, there appeared to be important variation in short sleep by industry for both Asians and Whites. For instance, there was an apparent decline in short sleep among Asians in the Accommodation and Food Industry that became significantly lower (p < 0.05) than Whites while short sleep remained generally stable in Whites over the study period. Short sleep prevalence estimates overlapped by race over time for the Manufacturing/Construction industry category. Short sleep was consistently higher in Asians than Whites over time in the Education and Healthcare industries, and the widest disparity over time was observed in the Finance Industry.

## Discussion

In this nationally representative study of Asians and Whites, we confirmed reports that short sleep duration is high in the US, but for the first time show that Asian Americans had an overall age-adjusted prevalence of short sleep that was higher than Whites. Furthermore, we show that the difference in short sleep prevalence between Asians and Whites varied importantly by both industry and occupation, with the largest gap observed in the Finance/information industry and among both professional and support services occupations. Our study, in combination with previous investigations, suggests that population patterns of sleep duration are likely influenced by a complex interplay between factors in the social and work environment [18,38]. Although a high prevalence of short sleep duration among manufacturing/construction, transportation/warehousing, and public administration workers was found in a prior study, the results of this study were not stratified by race. Racial/ethnic health disparities are likely influenced by occupational environments and stressors in the workplace that may, for example, affect sleep quantity and quality. Therefore, racial/ethnic differences in the work-sleep relationship deserve greater attention.

Prior research identifying risk factors for short sleep have focused on SES or race (confounded by SES) [17,39]. These studies identified that lower SES and Black race are significant risk factors for short sleep, and the relationships were presumed to reflect socioeconomic stressors, including the impact of discrimination on sleep. However, we recently showed that not considering race and SES (e.g. occupation) in combination may limit the inferences from such research. In particular, we recently showed that occupation significantly modified the associations between short sleep and race in a comparison of Blacks and Whites [23]. Similar to the results of that analysis, we now also show that Asian professionals have a higher prevalence of short sleep than White professionals.

Sociocultural factors may connect one’s job – a marker of socioeconomic position and potentially large source of psychosocial and environmental stressors – with their overall health as occupational characteristics influence specific sleep conditions. For instance, Asians may experience racial discrimination in the workplace and great pressure to succeed in professional environments, which can conceivably increase stress in ways that displace sleep [40-42]. The work-sleep relationship may also be affected by several factors including voluntary or involuntary extended work hours, rotating or shift work (albeit low in Asians) as well as stress related to the job [19,24,43-45]. A recent study using 2010 data from the National Health Interview Study found that whites (20.9 [20.0-22.0]) were more likely than Asians (16.6 [13.9-19.9]) to formally work at least 48 hours per week [46]. Although non-significant, it appeared that whites (8.1 [7.4-8.8]) were also slightly more likely to work at least 60 hours per week than Asians (5.9 [4.3-8.0]) as well as to engage in alternative shift work (28.1 [27.0-29.2]) for whites vs. 26.2 [22.8-29.8] for Asians). A similar proportion of whites (6.2 [5.6-6.8]) and Asians (6.7 [(5.0-8.8]) worked in temporary positions. Furthermore, technology (e.g. internet with email capabilities, cellular phones) may have also increased the accessibility of employees in ways that enhance job strain as well as disrupt sleep [47,48], and use of technology may have differential impacts by race/ethnicity. Acculturation and cultural factors (e.g. religious beliefs and practices, strong work ethic) may also be more unique sources of racial/ethnic differences in the work-sleep relationship. Additionally, the majority of Asians in this nationally representative sample were non-US born (74%), and the US born individuals appeared to have the shorter sleep, which is consistent with evidence that Western acculturation negatively influences sleep habits as has been observed among Mexican Americans [49]. As suggested by our subsidiary analysis, it would be useful to further explore sleep differences in those who were born in and outside of the US in addition to the impact of certain Asian ethnicities likely being overrepresented in certain occupations, which could spark additional research and ideas for intervention as it is apparent that short sleep may result from social, occupational, and behavioral factors.

The high prevalence of short sleep in Asians raises concerns that this factor may contribute to the risk of diabetes, hypertension, cardiovascular disease and other health problems in this group. Prior research has indicated that Asians report a low frequency of sleep complaints [17]. Unfortunately, there is a profound scarcity of data on sleep architecture and sleep disorders, such as sleep apnea, in Asian Americans [50]. Since some research suggests that short sleep associated with insomnia may have the most adverse effects on health [51,52] it would be important for future studies to further consider the influence of short sleep and sleep disturbances on specific health outcomes among Asian Americans.

Furthermore, since Asians overall tend to possess high SES and other favorable factors that may be protective against suboptimal sleep, there are important opportunities to better understand interactions between sleep duration and SES in studies of health outcomes across racial groups.

Our study has several limitations. For instance, our cross-sectional study design precluded our ability to investigate prospective associations between various industries of employment among the employed and sleep duration. We also relied solely on self-reported data. More objective measures of sleep duration than self-report can be obtained through polysomnography and actigraphy [53], but measurements from these technologies were unavailable. To our knowledge, there is also no available validation data on the quality of self-reported (compared to measured) sleep duration among Asian Americans, which presents an important topic for future research. Furthermore, we did not have data on sleep disorders or sleep quality. We also could not account for number of children in the household, which likely influences sleep and differs by race. We also did not have access to data on medication use that may affect sleepiness. Employment status, which can be more variable for lower-SES, minority groups, was based on participants being employed during the week prior to the interview [54]; however, we do not expect for employment status to be more highly variable in Asians compared to Whites. Shift work, shown to differ by race and increase risk of disease, could not be accounted for although we do not expect Asians and Whites to have different participation levels of shift work [24,55,56]. Additionally, we did not have enough statistical power to test for differences among the various Asian-American groups, despite their known heterogeneity. For instance, Japanese Americans have the highest SES of any group in the US, but Vietnamese have the lowest SES [57].

Nonetheless, our study has important strengths that contribute to the literature. For instance, our data were based on a large population of Asian Americans for which data is typically sparse. We were also able to stratify by multiple factors (e.g. race/ethnicity, industry) while providing stable, robust estimates. Furthermore, we had access to 8 successive years of sleep data, enhancing our power to investigate sleep disparities and trends. These data are also nationally representative and were recently collected. Lastly, prevalence ratios were directly estimated, which makes it easier to interpret the results compared to odds ratios.

## Conclusion

Asian-White differences in short sleep duration varied importantly by industry of employment and occupation, and these complex differences reflect the need to identify as well as understand sociocultural factors that may influence the work-sleep relationship in hopes of effectively addressing the identified sleep disparities for optimal health and productivity among workers in the US.

## Competing interests

Authors have no conflicts of interest to disclose.

## Authors’ contributions

CLJ, IK, SR and FBH study concept and design. CLJ acquisition of data. CLJ statistical analysis. CLJ, IK, SR and FBH interpretation of data. CLJ drafting of the manuscript. CLJ, IK, SR, HSJ and FBH critical revision of the manuscript for important intellectual content. IK and FBH administrative, technical, and material support. IK and FBH obtaining funding and study supervision. All authors read and approved the final manuscript.

## Pre-publication history

The pre-publication history for this paper can be accessed here:

http://www.biomedcentral.com/1471-2458/14/552/prepub

## Supplementary Material

Additional file 1: Table S1 Sociodemographic, Health Behavior, and Clinical Characteristics among NHIS Participants with Short Sleep Duration by Race/ethnicity, 2004-2011 (N=35,961). Click here for file

Additional file 2: Table 2 Adjusted Prevalence Ratios of Short Sleep Duration for Asians Compared to Whites Born in the US (n=114,177) and not (n=11,380) by Industry of Employment, National Health Interview Survey, 2004-2011. Click here for file

## References

[B1] Buxton OM Marcelli E Short and long sleep are positively associated with obesity, diabetes, hypertension, and cardiovascular disease among adults in the United States Soc Sci Med 2010 71 5 1027 1036 10.1016/j.socscimed.2010.05.041 20621406

[B2] Hammond EC Some preliminary findings on physical complaints from a prospective study of 1,064,004 Men and women Am J Public Health Nations Health 1964 54 11 23 14117648 10.2105/ajph.54.1.11 PMC1254627

[B3] Gangwisch JE Heymsfield SB Boden-Albala B Buijs RM Kreier F Opler MG Pickering TG Rundle AG Zammit GK Malaspina D Sleep duration associated with mortality in elderly, but not middle-aged, adults in a large US sample Sleep 2008 31 8 1087 1096 18714780 PMC2542954

[B4] Gangwisch JE Heymsfield SB Boden-Albala B Buijs RM Kreier F Pickering TG Rundle AG Zammit GK Malaspina D Sleep duration as a risk factor for diabetes incidence in a large U.S. sample Sleep 2007 30 12 1667 1673 18246976 10.1093/sleep/30.12.1667 PMC2276127

[B5] Gottlieb DJ Punjabi NM Newman AB Resnick HE Redline S Baldwin CM Nieto FJ Association of sleep time with diabetes mellitus and impaired glucose tolerance Arch Intern Med 2005 165 8 863 867 10.1001/archinte.165.8.863 15851636

[B6] Gottlieb DJ Redline S Nieto FJ Baldwin CM Newman AB Resnick HE Punjabi NM Association of usual sleep duration with hypertension: the Sleep Heart Health Study Sleep 2006 29 8 1009 1014 16944668 10.1093/sleep/29.8.1009

[B7] Alvarez GG Ayas NT The impact of daily sleep duration on health: a review of the literature Prog Cardiovasc Nurs 2004 19 2 56 59 10.1111/j.0889-7204.2004.02422.x 15133379

[B8] Ayas NT White DP Al-Delaimy WK Manson JE Stampfer MJ Speizer FE Patel S Hu FB A prospective study of self-reported sleep duration and incident diabetes in women Diabetes Care 2003 26 2 380 384 10.2337/diacare.26.2.380 12547866

[B9] Steptoe A Peacey V Wardle J Sleep duration and health in young adults Arch Intern Med 2006 166 16 1689 1692 10.1001/archinte.166.16.1689 16983045

[B10] Taheri S Lin L Austin D Young T Mignot E Short sleep duration is associated with reduced leptin, elevated ghrelin, and increased body mass index PLoS Med 2004 1 3 e62 10.1371/journal.pmed.0010062 15602591 PMC535701

[B11] Grandner MA Hale L Moore M Patel NP Mortality associated with short sleep duration: the evidence, the possible mechanisms, and the future Sleep Med Rev 2010 14 3 191 203 10.1016/j.smrv.2009.07.006 19932976 PMC2856739

[B12] Liu R Zee PC Chervin RD Arguelles LM Birne J Zhang S Christoffel KK Brickman WJ Zimmerman D Wang B Wang G Xu X Wang X Short sleep duration is associated with insulin resistance independent of adiposity in Chinese adult twins Sleep Med 2011 12 9 914 919 10.1016/j.sleep.2011.04.006 21940204 PMC3210935

[B13] Yea H Relation between sleep quality and quantity, quality of life, and risk of developing diabetes in healthy workers in Japan: the High-risk and Population Strategy for Occupational Health Promotion (HIPOP-OHP) Study BMC Public Health 2007 7 1 129 10.1186/1471-2458-7-129 17597542 PMC1924854

[B14] Beckles GL Zhu J Moonesinghe R Diabetes - United States, 2004 and 2008 MMWR Surveill Summ 2011 60 Suppl 90 93 21430631

[B15] Li KK Powell NB Kushida C Riley RW Adornato B Guilleminault C A comparison of Asian and white patients with obstructive sleep apnea syndrome Laryngoscope 1999 109 12 1937 1940 10.1097/00005537-199912000-00007 10591350

[B16] Cappuccio FP D’Elia L Strazzullo P Miller MA Sleep duration and all-cause mortality: a systematic review and meta-analysis of prospective studies Sleep 2010 33 5 585 592 20469800 10.1093/sleep/33.5.585 PMC2864873

[B17] Grandner MA Patel NP Gehrman PR Xie D Sha D Weaver T Gooneratne N Who gets the best sleep? Ethnic and socioeconomic factors related to sleep complaints Sleep Med 2010 11 5 470 478 10.1016/j.sleep.2009.10.006 20388566 PMC2861987

[B18] Luckhaupt SE Tak S Calvert GM The prevalence of short sleep duration by industry and occupation in the National Health Interview Survey Sleep 2010 33 2 149 159 20175398 10.1093/sleep/33.2.149 PMC2817902

[B19] Kuhn P Lozano F The expanding workweek? understanding trends in long work hours among U.S. Men, 1979–2006 J Labor Econ 2008 26 2 311 343 10.1086/533618

[B20] Karasek RA Theorell T Healthy work: stress, productivity, and the reconstruction of working life 1992 New York, New York: Basic books, Inc

[B21] Krieger N Waterman PD Hartman C Bates LM Stoddard AM Quinn MM Sorensen G Barbeau EM Social hazards on the job: workplace abuse, sexual harassment, and racial discrimination–a study of Black, Latino, and White low-income women and men workers in the United States Int J Health Serv 2006 36 1 51 85 10.2190/3EMB-YKRH-EDJ2-0H19 16524165

[B22] Grandner MA Hale L Jackson N Patel NP Gooneratne NS Troxel WM Perceived racial discrimination as an independent predictor of sleep disturbance and daytime fatigue Behav Sleep Med 2012 10 4 235 249 10.1080/15402002.2012.654548 22946733 PMC3434973

[B23] Jackson CL Redline S Kawachi I Williams MA Hu FB Racial disparities in short sleep duration by occupation and industry Am J Epidemiol 2013 178 9 1442 1451 10.1093/aje/kwt159 24018914 PMC3888251

[B24] Pilcher JJ Lambert BJ Huffcutt AI Differential effects of permanent and rotating shifts on self-report sleep length: a meta-analytic review Sleep 2000 23 2 155 163 10737332

[B25] Presser H Race-ethnic and gender differences in nonstandard work shifts Work Occup 2003 30 412 439 10.1177/0730888403256055

[B26] Tomfohr L Pung MA Edwards KM Dimsdale JE Racial differences in sleep architecture: the role of ethnic discrimination Biol Psychol 2012 89 1 34 38 10.1016/j.biopsycho.2011.09.002 21925567 PMC3245778

[B27] Hughes D Dodge MA African American women in the workplace: relationships between job conditions, racial bias at work, and perceived job quality Am J Community Psychol 1997 25 5 581 599 10.1023/A:1024630816168 9485575

[B28] James SA John Henryism and the health of African-Americans Cult Med Psychiatry 1994 18 2 163 182 10.1007/BF01379448 7924399

[B29] National Center for Health Statistics, Centers for Disease Control and Prevention National Health Interview Survey. Hyattsville, MD. Available at: http://www.cdc.gov/nchs/nhis.htm. Accessed November, 2013

[B30] Voss U Tuin I Integration of immigrants into a new culture is related to poor sleep quality Health Qual Life Outcomes 2008 6 61 10.1186/1477-7525-6-61 18691437 PMC2518135

[B31] Jackson CL Redline S Kawachi I Hu FB Association between sleep duration and diabetes in black and white adults Diabetes Care 2013 36 11 3557 3565 10.2337/dc13-0777 24026552 PMC3816913

[B32] Wang J Thornton JC Russell M Burastero S Heymsfield S Pierson RN Jr Asians have lower body mass index (BMI) but higher percent body fat than do whites: comparisons of anthropometric measurements Am J Clin Nutr 1994 60 1 23 28 8017333 10.1093/ajcn/60.1.23

[B33] Minnesota Population Center and State Health Access Data Assistance Center, Integrated Health Interview Series: Version 3.0 2010 Minneapolis: University of Minnesota

[B34] Wolters KM Introduction to variance estimation 1990 New York, NY: Springer-Verlag

[B35] Rao JN Scott AJ A simple method for the analysis of clustered binary data Biometrics 1992 48 2 577 585 10.2307/2532311 1637980

[B36] Stata Corp Stata TX, 2007. 2008. Statistical Software: Released 10 2010 College Station: Stata Corporation

[B37] Barros AJ Hirakata VN Alternatives for logistic regression in cross-sectional studies: an empirical comparison of models that directly estimate the prevalence ratio BMC Med Res Methodol 2003 3 21 10.1186/1471-2288-3-21 14567763 PMC521200

[B38] Adler NE Newman K Socioeconomic disparities in health: pathways and policies Health Aff (Millwood) 2002 21 2 60 76 10.1377/hlthaff.21.2.60 11900187

[B39] Patel NP Grandner MA Xie D Branas CC Gooneratne N “Sleep disparity” in the population: poor sleep quality is strongly associated with poverty and ethnicity BMC Public Health 2010 10 475 10.1186/1471-2458-10-475 20701789 PMC2927542

[B40] Bhattacharya G Schoppelrey SL Preimmigration beliefs of life success, postimmigration experiences, and acculturative stress: South Asian immigrants in the United States J Immigr Health 2004 6 2 83 92 15014225 10.1023/B:JOIH.0000019168.75062.36

[B41] Liang CT Fassinger RE The role of collective self-esteem for Asian Americans experiencing racism-related stress: a test of moderator and mediator hypotheses Cultur Divers Ethnic Minor Psychol 2008 14 1 19 28 18229997 10.1037/1099-9809.14.1.19

[B42] Osajima K Asian Americans as the model minority: an analysis of the popular press image in the 1960s and Companion Asian Am Stud 1980 2005 215 225

[B43] Tucker P Smith L Macdonald I Folkard S The impact of early and late shift changeovers on sleep, health, and well-being in 8- and 12-hour shift systems J Occup Health Psychol 1998 3 3 265 275 9684216 10.1037//1076-8998.3.3.265

[B44] Ota A Masue T Yasuda N Tsutsumi A Mino Y Ohara H Association between psychosocial job characteristics and insomnia: an investigation using two relevant job stress models–the demand-control-support (DCS) model and the effort-reward imbalance (ERI) model Sleep Med 2005 6 4 353 358 10.1016/j.sleep.2004.12.008 15978518

[B45] Ruggiero JS Redeker NS Effects of napping on sleepiness and sleep-related performance deficits in night-shift workers: a systematic review Biol Res Nurs 2014 16 2 134 142 Epub 2013 Feb 13 10.1177/1099800413476571 23411360 PMC4079545

[B46] Alterman T Luckhaupt SE Dahlhamer JM Ward BW Calvert GM Prevalence rates of work organization characteristics among workers in the U.S.: data from the 2010 National Health Interview Survey Am J Ind Med 2013 56 6 647 659 10.1002/ajim.22108 22911666 PMC4557703

[B47] Costa G The 24-hour society between myth and reality J Hum Ergol 2001 30 1–2 15 20 14564852

[B48] Presser HB Towards a 24-hour economy Science 1999 284 1777 1779

[B49] Seicean S Neuhauser D Strohl K Redline S An exploration of differences in sleep characteristics between Mexico-born US immigrants and other Americans to address the Hispanic Paradox Sleep 2011 34 8 1021 1031 21804664 10.5665/SLEEP.1154 PMC3138157

[B50] Mirrakhimov AE Sooronbaev T Mirrakhimov EM Prevalence of obstructive sleep apnea in Asian adults: a systematic review of the literature BMC Pulm Med 2013 13 10 10.1186/1471-2466-13-10 23433391 PMC3585751

[B51] Vgontzas AN Liao D Pejovic S Calhoun S Karataraki M Bixler EO Insomnia with objective short sleep duration is associated with type 2 diabetes: a population-based study Diabetes Care 2009 32 11 1980 1985 10.2337/dc09-0284 19641160 PMC2768214

[B52] Fernandez-Mendoza J Vgontzas AN Liao D Shaffer ML Vela-Bueno A Basta M Bixler EO Insomnia with objective short sleep duration and incident hypertension: the Penn State Cohort Hypertension 2012 60 4 929 935 10.1161/HYPERTENSIONAHA.112.193268 22892811 PMC3679545

[B53] Lauderdale DS Knutson KL Yan LL Liu K Rathouz PJ Self-reported and measured sleep duration: how similar are they? Epidemiology 2008 19 6 838 845 10.1097/EDE.0b013e318187a7b0 18854708 PMC2785092

[B54] Muntaner C Hadden WC Kravets N Social class, race/ethnicity and all-cause mortality in the US: longitudinal results from the 1986–1994 National Health Interview Survey Eur J Epidemiol 2004 19 8 777 784 15469035 10.1023/b:ejep.0000036569.39399.68

[B55] Ohayon MM Smolensky MH Roth T Consequences of shiftworking on sleep duration, sleepiness, and sleep attacks Chronobiol Int 2010 27 3 575 589 10.3109/07420521003749956 20524802

[B56] Ertel KA Berkman LF Buxton OM Socioeconomic status, occupational characteristics, and sleep duration in African/Caribbean immigrants and US White health care workers Sleep 2011 34 4 509 518 21461330 10.1093/sleep/34.4.509 PMC3065262

[B57] Frisbie WP Cho Y Hummer RA Immigration and the health of Asian and Pacific Islander adults in the United States Am J Epidemiol 2001 153 4 372 380 10.1093/aje/153.4.372 11207155

