# Supplementary material for: Asian-White disparities in short sleep duration by industry of employment and occupation in the US: a cross-sectional study
Source: BMC Public Health. 2014 Jun 3;14:552. doi: 10.1186/1471-2458-14-552 (PMC4057824; doi:10.1186/1471-2458-14-552)
Supplement: Additional file 1: Table S1 — Sociodemographic, Health Behavior, and Clinical Characteristics among NHIS Participants with Short Sleep Duration by Race/ethnicity, 2004-2011 (N=35,961). [file 1471-2458-14-552-S1.doc]

**Additional file 1: Table S1. Sociodemographic, Health Behavior, and Clinical**

**Characteristics among NHIS Participants with Short Sleep Duration by**

**Race/ethnicity, 2004-2011 (N=35,961)**

|  | **Short Sleep (<7 hours)** | |
| --- | --- | --- |
|  | **White** | **Asian** |
| **Sample size, N (%)** | 33,354 (94) | 2,607 (6) |
| **Age, years ± SE** | 49.0 ± 0.11 | 47.1 ± 0.33 |
| **Age group, (%)** |  |  |
| 18-49 | 54 | 59 |
| 50-64 | 31 | 29 |
| ≥65 | 15 | 12 |
| **Women** | 48 | 47 |
| **Educational attainment** |  |  |
| <High school | 32 | 17 |
| High school graduate | 9 | 7 |
| Some college | 34 | 25 |
| ≥ College | 25 | 51 |
| **Non-US born** | 26 | 74 |
| **Marital status** |  |  |
| Married | 57 | 66 |
| Divorced/separated/widowed | 23 | 12 |
| Never married | 20 | 21 |
| **Living in poverty** | 10 | 9 |
| **Class of worker** |  |  |
| Private wage | 75 | 78 |
| Government | 16 | 14 |
| Self employed | 9 | 8 |
| **Occupation** |  |  |
| Professional/management | 19 | 29 |
| Support Services | 44 | 45 |
| Laborers | 37 | 26 |
| **Industry** |  |  |
| Manufacturing/Construction | 33 | 24 |
| Retail | 12 | 10 |
| Finances/Information | 9 | 11 |
| Profess/ Admin/Man | 9 | 13 |
| Education | 8 | 8 |
| Heath care | 12 | 16 |
| Accommodation and Food | 6 | 6 |
| Public Administration, Arts | 11 | 12 |
| **Health behaviors** |  |  |
| Smoking status |  |  |
| Never | 47 | 72 |
| Current | 25 | 15 |
| Former | 28 | 13 |
| Alcohol consumption |  |  |
| Never | 13 | 35 |
| Current | 70 | 54 |
| Former | 17 | 11 |
| Leisure-time physical activity |  |  |
| Never/unable | 33 | 34 |
| Low | 34 | 36 |
| High | 33 | 30 |
| **Clinical characteristics** |  |  |
| Overweight/Obese* | 67 | 67 |
| Obese* | 31 | 28 |
| Hypertension (yes) | 31 | 26 |
| Diabetes (yes) | 8 | 8 |
| **Health status** |  |  |
| Excellent/very good | 58 | 61 |
| Good | 27 | 29 |
| Fair/poor | 15 | 10 |
| **Region of country** |  |  |
| Northeast | 20 | 19 |
| Midwest | 29 | 14 |
| South | 34 | 20 |
| West | 17 | 46 |

Weighted estimates; n (%) or mean ± SE; SE=standard error

Overweight/Obese defined by Body Mass Index ≥25 kg/m**^2^**;

Obesity defined by Body Mass Index ≥30 kg/m**^2^**

* Asian BMI cut point for overweight=23 kg/m^2^ and obese=27 kg/m^2^
