# Supplementary material for: Asian-White disparities in short sleep duration by industry of employment and occupation in the US: a cross-sectional study
Source: BMC Public Health. 2014 Jun 3;14:552. doi: 10.1186/1471-2458-14-552 (PMC4057824; doi:10.1186/1471-2458-14-552)
Supplement: Additional file 2: Table 2 — Adjusted Prevalence Ratios of Short Sleep Duration for Asians Compared to Whites Born in the US (n=114,177) and not (n=11,380) by Industry of Employment, National Health Interview Survey, 2004-2011. [file 1471-2458-14-552-S2.doc]

**Additional file 2: Table S2. Adjusted Prevalence Ratios of Short Sleep Duration for Asians Compared to Whites Born in the US (n=114,177) and not (n=11,380) by Industry of Employment, National Health Interview Survey, 2004-**2011

|  | **US-born** | | **Non-US born** | |
| --- | --- | --- | --- | --- |
|  | **INDUSTRY** | | | |
| **Manufacturing/Construction** | 34,111  (434 Asians;  33,677 Whites) | 1.30  (1.08-1.58) | 2,770  (1,434 Asians; 1,336 Whites) | 1.08  (0.91-1.29) |
| **Retail** | 12,686  (194 Asians;  12,492 Whites) | 1.13  (0.82-1.57) | 1,179  (622 Asians;  557 Whites) | 1.05  (0.78-1.42) |
| **Finances/Information** | 11,230  (256 Asians;  10,974 Whites) | 1.57  (1.26-1.96) | 1,089  (574 Asians; 515 Whites) | 1.37  (1.04-1.79) |
| **Profess/ Admin/Man** | 10,814  (273 Asians;  10,541 Whites) | 1.20  (0.92-1.56) | 1,517  (923 Asians; 594 Whites) | 1.17  (0.90-1.53) |
| **Education** | 11,795  (270 Asians;  11,795 Whites) | 1.37  (1.05-1.79) | 1,127  (585 Asians;  542 Whites) | 1.30  (0.92-1.84) |
| **Health care and Social Services** | 13,639  (246 Asians;  13,393 Whites) | 1.51  (1.17-1.96) | 1,501  (884 Asians; 617 Whites) | 1.34  (1.05-1.70) |
| **Accommodation and Food** | 6,073  (131 Asians;  5,942 Whites) | 0.82  (0.54-1.24) | 858  (541 Asians; 317 Whites) | 0.84  (0.59-1.20) |
| **Public Administration, Arts** | 13,829  (302 Asians;  13,527 Whites) | 1.38  (1.07-1.77) | 1,339  (706 Asians;  633 Whites) | 1.13  (0.87-1.47) |
| **OCCUPATION** | | | | |
| **Professional/Management** | 23,619  (594 Asians;  23,025 Whites) | 1.56  (1.33-1.83) | 3,215  (1,943 Asians; 1,272 Whites) | 1.12  (0.93-1.36) |
| **Support Services** | 54,166  (1,057 Asians;  53,109 Whites) | 1.28  (1.09-1.50) | 5,084  (2,724 Asians;  2,360 Whites) | 1.32  (1.12-1.55) |
| **Laborers** | 35,979  (443 Asians;  35,536 Whites) | 1.01  (0.79-1.30) | 3,018  (1,456 Asians; 1,562 Whites) | 1.08  (0.89-1.31) |

Industry and occupation adjustment: age categories, sex, marital status, educational attainment, smoking status,

alcohol consumption, physical activity, health status, body mass index, hypertension, diabetes, heart disease,

cancer, class of occupation, occupation (except for occupation adjustment), living in poverty, and household income
